# Cost-effectiveness analysis of first-line combination chemotherapy regimens for metastatic pancreatic cancer and evidence-based pricing strategy of liposomal irinotecan in China

**DOI:** 10.3389/fphar.2024.1488645

**Published:** 2024-12-20

**Authors:** Zuojuan Xiang, Ling Ma, Zhengxiong Li, Yingzhou Fu, Yong Pan

**Affiliations:** ^1^ Department of Pharmacy, The Affiliated Cancer Hospital of Xiangya School of Medicine, Central South University, Hunan Cancer Hospital, Changsha, China; ^2^ Department of Clinical pharmacy, The First People’s Hospital of Yunnan Province, The Affiliated Hospital of Kunming University of Science and Technology, Kunming, China; ^3^ School of Medical Informatics and Engineering, Xuzhou Medical University, Xuzhou, China

**Keywords:** metastatic pancreatic cancer, cost-effectiveness, FOLFIRINOX, NALIRIFOX, GEMNABP, liposomal irinotecan

## Abstract

**Background:**

The phase III NAPOLI-3 trial, which upgraded FOLFIRINOX (leucovorin, fluorouracil, irinotecan and oxaliplatin) to NALIRIFOX (liposomal irinotecan, oxaliplatin, leucovorin, and fluorouracil), demonstrated the superiority of NALIRIFOX over GEMNABP (gemcitabine and nab-paclitaxel) as the first-line treatment for metastatic pancreatic ductal adenocarcinoma. The purpose of this study was to assess the cost-effectiveness of NALIRIFOX, FOLFIRINOX, and GEMNABP, and to simulate the price of liposomal irinotecan at which NALIRIFOX could achieve cost-effectiveness.

**Methods:**

A partitioned survival model was performed to evaluate the cost-effectiveness of NALIRIFOX, FOLFIRINOX and GEMNABP from the perspective of the Chinese healthcare system. Survival data was obtained from a recently published network meta-analysis (NMA). Drug prices were collected from the database of the Hunan Province Drug and Medical Consumables Procurement Management Subsystem. Other cost and utility values were sourced from established literature. Cumulative costs, LYs (life-years), quality-adjusted life years (QALYs), incremental cost-effectiveness ratios (ICERs), net monetary benefits (NMBs) and incremental net monetary benefits (INMBs) were the main outputs. Furthermore, the variations in ICER were analyzed as the price of liposomal irinotecan gradually decreased when comparing NALIRIFOX with FOLFIRINOX or GEMNABP. The robustness of the model was assessed by sensitivity analysis and scenario analysis.

**Results:**

At the willingness-to-pay (WTP) threshold of $38,223.34, GEMNABP was the favored treatment. NALIRIFOX was associated with the highest LYs, QALYs, and cost. The cost-effectiveness of NALIRIFOX would be obtained if the price of liposomal irinotecan was less than $3.36/mg and $2.08/mg compared to FOLFIRINOX and GEMNABP, respectively, without considering the patient assistance program (PAP). Sensitivity analysis and scenario analysis revealed that the results of the model were stable.

**Conclusion:**

From an economic standpoint, GEMNABP represents the favored choice in the prevailing market conditions among these three first-line combination chemotherapy regimens. The price simulation of liposomal irinotecan conducted in this study could provide valuable evidence for healthcare decision-making. Further evidence regarding the budget impact is still needed.

## 1 Introduction

Pancreatic cancer, a highly malignant digestive system cancer, poses a serious threat to human health. It ranks twelfth in the world in terms of newly diagnosed cases among all malignant tumors, but ranks sixth in terms of mortality rate (Bray et al., 2024). In China, the estimated number of new confirmed cases was 118,700, and the number of deaths was 106,300 in 2022 (Han et al., 2024). Pancreatic cancer typically onsets insidiously and progresses rapidly, leading to a diagnosis at an advanced stage for most patients. Less than 20% of patients have the chance for surgical intervention, and the 5-year survival rate for patients with metastatic pancreatic cancer is a mere 3% (Wainberg et al., 2023; Golivi et al., 2024).

The dense stroma matrix surrounding pancreatic cancer cells hinders the delivery of therapeutic drugs and immune cells to the target site, leading to diminished drug effectiveness (Luo and Zhang, 2024). The targeted and immunotherapy approaches, which have made significant strides in the treatment of other types of tumors, have demonstrated poor efficacy when applied to pancreatic cancer (Liu et al., 2022). For patients with metastatic pancreatic cancer, gemcitabine monotherapy was proved to be more effective than fluorouracil (Burris et al., 1997). Therefore, in the past two decades, clinicians have endeavored to identify drugs or combination therapy regimens capable of surpassing the effectiveness of gemcitabine. In this context, the combination of leucovorin, fluorouracil, irinotecan and oxaliplatin (FOLFIRINOX) (Conroy et al., 2011) and the combination of gemcitabine and nab-paclitaxel (GEMNABP) (Von Hoff et al., 2013) have demonstrated favorable outcomes in clinical trials, establishing them as the gold standard first-line chemotherapy regimens. Recently, the phase III NAPOLI-3 clinical trial upgraded FOLFIRINOX to NALIRIFOX, which included liposomal irinotecan, oxaliplatin, leucovorin, and fluorouracil. The findings indicated that both progression-free survival (PFS) and overall survival (OS) of NALIRIFOX were superior to GEMNABP (Wainberg et al., 2023). Based on the positive results, NALIRIFOX has been recommended as a new first-line treatment for metastatic pancreatic cancer in the 2023 edition of National Comprehensive Cancer Network (NCCN) guidelines and the 2024 edition of Chinese Society of Clinical Oncology (CSCO) guidelines.

Only NALIRIFOX and GEMNABP were head-to-head compared in the NAPOLI-3 trial, and to date, neither of these two regimens has been directly compared with FOLFIRINOX. Recently, Nichetti F et al. conducted a network meta-analysis (NMA) comparing these three treatments in terms of PFS, OS, and toxicity (Nichetti et al., 2024). The results revealed that the median PFS for NALIRIFOX and FOLFIRINOX was 7.4 months and 7.3 months, respectively, showing no significant difference. In contrast, GEMNABP demonstrated a significantly poorer median PFS of 5.7 months. Similarly, the OS of NALIRIFOX and FOLFIRINOX was 11.7 months and 11.1 months, respectively, whereas GEMNABP displayed a poorer OS of 10.4 months. In comparison to the other two treatments, NALIRIFOX was linked to a lower occurrence of grade 3 or higher hematological toxicity. However, it exhibited a higher risk of severe diarrhea compared to GEMNABP.

Clinical data serves as a critical basis for the evidence-based use of drugs in clinical practice. However, to the best of our knowledge, there is a dearth of pharmacoeconomic evaluations of these three treatments, which are also essential for promoting rational drug use. Although NALIRIFOX has demonstrated favorable clinical outcomes, its use involves a relatively expensive drug, liposomal irinotecan, which encapsulates irinotecan in pegylated liposomal particles to achieve improved pharmacokinetics and has been approved for metastatic pancreatic cancer (Melisi et al., 2024). In fact, among the drugs used in these three treatments, only liposomal irinotecan has not been included in the Chinese Basic Medical Insurance Drug List. Therefore, drawing from the NMA, this study conducted a cost-effectiveness assessment of these three regimens from the perspective of the Chinese healthcare system and a price simulation of liposomal irinotecan to inform the pricing strategy. It aimed to provide decision-makers with valuable references for optimizing the allocation of healthcare resources and offering doctors important economic evidence for selecting appropriate chemotherapy regimen for their patients.

## 2 Methods

This cost-effectiveness analysis was conducted according to Consolidated Health Economic Evaluation Reporting Standards (CHEERS) and from the perspective of Chinese healthcare system (Supplementary Table S1). Total costs, LYs (life-years), QALYs (quality-adjusted life-years), incremental cost-effectiveness ratios (ICERs), net monetary benefits (NMBs) and incremental net monetary benefits (INMBs) were the main outputs. According to the 2020 version of the China Guidelines for Pharmacoeconomic Evaluations (CGPE), whether cost-effectiveness of the treatment was determined by comparing the ICER value with the willingness to pay (WTP) threshold of $38,223.34, which was three times the gross domestic product (GDP) *per capita* of China in 2022, or by evaluating the INMB. INMB = WTP*(E2 − E1) − (C2 − C1). INMB >0 indicates cost-effective.

### 2.1 Patient population and regimen

This model was conducted based on the NMA, which involved the reconstruction of individual patient data from 7 phase III clinical trials (Conroy et al., 2011; Von Hoff et al., 2013; Philip et al., 2019; Van Cutsem et al., 2020; Tempero et al., 2021; Bekaii-Saab et al., 2023; Wainberg et al., 2023). Aligned with the NMA, the hypothetical cohort in this study comprised patients with metastatic pancreatic ductal adenocarcinoma who received NALIRIFOX, FOLFIRINOX, or GEMNABP as their first-line treatment planned at standard dose intensity. Prior adjuvant treatment was allowed. Patients were adults aged 18 years or older, with an Eastern Cooperative Oncology Group performance status score (ECOG PS) of 0 or 1.44% of patients were male. Since this study was based on previously published data and did not involve patient recruitment or a retrospective analysis of primary patient data, ethical approval was not required.

All medications involved in this model were administered intravenously. Specific dosing regimens and doses were collected from the corresponding clinical trials and detailed in the Table 1. When determining the dosage, an average body surface area of 1.72 m^2^ was used (Qiao et al., 2021).

**TABLE 1:** Specific dosing regimens.

First-line chemotherapy regimen	Dosing schemes	Second-line chemotherapy regimen	Dosing schemes
NALIRIFOX	Liposomal irinotecan 50 mg/m^2^ + oxaliplatin 60 mg/m^2^ + LV 400 mg/m^2^ +fluorouracil 2,400 mg/m^2^ over 46 h; every 14 days	GEMNABP	GEM 1000 mg/m^2^ + NABP 125 mg/m^2^; Days 1, 8, 15, and every 28 days
FOLFIRINOX	Irinotecan 180 mg/m^2^ + oxaliplatin 85 mg/m^2^ + LV 400 mg/m^2^ +fluorouracil bolus 400 mg/m^2^ +2,400 mg/m^2^ over 46 h; every 14 days	GEMNABP	GEM 1000 mg/m^2^ + NABP 125 mg/m^2^; Days 1, 8, 15, and every 28 days
GEMNABP	GEM 1000 mg/m^2^ + NABP 125 mg/m^2^; Days 1, 8, 15, and every 28 days	FOLFOX	Oxaliplatin 85 mg/m^2^ on day 1 +LV 200 mg/m^2^ +2,400 mg/m^2^ over 46 h every 14 days

GEM, gemcitabine; LV, leucovorin; NABP, nab-paclitaxel.

### 2.2 Model structure

**FIGURE 1:**
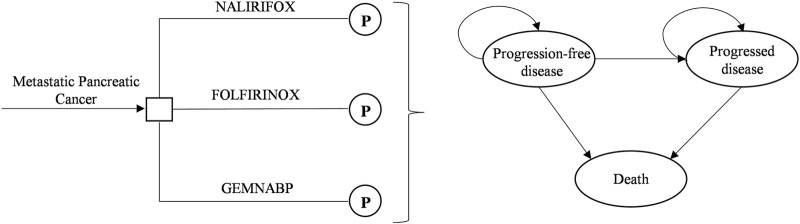
Model structure. P, partitioned survival model.

We employed the TreeAge Pro 2019 software to construct a partitioned survival model comprising three distinct health states: PFS, progressed disease (PD), and death. The model structure was shown in Figure 1. The partitioned survival model calculates the proportion of patients in different health states based on the area under the OS and PFS curves. This approach yields results that closely approximate the actual observed data. Given the highly aggressive nature of metastatic pancreatic cancer, the time horizon for the model was established at 5 years, within which 99% of the patients in the model had died. The cycle length was set at 28 days to align with the dosing regimen.

Survival data used in this model were derived from the reconstructed and validated survival curves in the NMA. WebPlotDigitizer was used to extract PFS and OS data points from the Kaplan-Meier curves, followed by curve reconstruction using the R software (version 4.3.2). As shown in Supplementary Figure S1 and Supplementary Table S2, the median PFS and median OS values of the reconstructed curves were very close to the original ones. Based on Guyot et al.'s algorithm, we extrapolated survival curves and used exponential, Weibull, log-normal, log-logistic, Gompertz, and generalized gamma parametric survival functions to fit the survival data. These standard parametric models are the most commonly used method in existing studies, despite their potential limitations in capturing survival curve inflection points compared to more flexible parametric models. However, given the nature of the traditional chemotherapy drugs involved in this study, which were less likely to lead to plateaus or other complex scenarios in survival curves, and because the survival curves were relatively mature, the use of these standard parametric models was considered appropriate. The goodness of fit of the model was assessed using the Akaike Information Criterion (AIC) and the Bayesian Information Criterion (BIC). Therefore, we ultimately fitted the OS curve of FOLFIRINOX with a Weibull distribution, and the remaining survival curves with a Gamma distribution, based on the lowest AIC and BIC values and consistency with visual inspection. This approach ensured that the chosen distributions provided the optimal fit to the original curves (Supplementary Figure S2; Supplementary Table S3). Key parameters of the optimal distribution of survival curves were detailed in Table 2. Following the 2020 edition of CGPE, both cost and utility values were discounted at a rate of 5% in this model.

**TABLE 2:** Key parameters.

Parameters	Baseline value	Minimum	Maximum	Distribution	References
Kaplan Meier survival curve					
NALIRIFOX-OS	Shape 1.4047, rate 0.1006	-	-	Gamma	Nichetti et al. (2024)
NALIRIFOX-PFS	Shape 1.3473, rate 0.1479	-	-	Gamma	Nichetti et al. (2024)
FOLFIRINOX-OS	Shape 1.3484, scale 0.0261	-	-	Weibull	Nichetti et al. (2024)
FOLFIRINOX-PFS	Shape 1.5054, rate 0.1730	-	-	Gamma	Nichetti et al. (2024)
GEMNABP-OS	Shape 1.6851, rate 0.1311	-	-	Gamma	Nichetti et al. (2024)
GEMNABP-PFS	Shape 1.7450, rate 0.2355	-	-	Gamma	Nichetti et al. (2024)
Cost ($)					
Biochemical and blood routine examination/cycle	29.21	23.37	35.05	Gamma	Li et al. (2021)
Radiological examination/cycle	95.53	76.42	114.64	Gamma	Li et al. (2021)
Best supportive care	118.29	94.63	141.95	Gamma	Li et al. (2021)
Terminal care	1,967.95	1,574.36	2,361.54	Gamma	Li et al. (2021)
Cost of drugs					
Liposomal irinotecan/1 mg	24.88	19.90	29.86	Gamma	Local charge
Irinotecan/100 mg	10.01	8.01	12.01	Gamma	Local charge
Oxaliplatin/100 mg	65.13	52.10	78.16	Gamma	Local charge
Leucovorin/100 mg	2.43	1.94	2.92	Gamma	Local charge
Fluorouracil/100 mg	3.28	2.62	3.94	Gamma	Local charge
Gemcitabine/1000 mg	5.01	4.01	6.01	Gamma	Local charge
Nab-paclitaxel/100 mg	22.24	17.79	26.69	Gamma	Local charge
Cost of grade ≥3 AEs					
Anemia	42.42	33.94	50.90	Gamma	Cui et al. (2021)
Neutrophil count decreased	113.32	90.66	135.98	Gamma	Cui et al. (2021)
Febrile neutropenia	279.40	223.52	335.28	Gamma	Cui et al. (2021)
Platelet count decreased	378.48	302.78	454.18	Gamma	Cui et al. (2021)
Diarrhea	5.10	4.08	6.12	Gamma	Cui et al. (2021)
Peripheal neuropathy	58.76	47.01	70.51	Gamma	Cui et al. (2021)
Vomit	28.33	22.66	34.10	Gamma	Cui et al. (2021)
Fatigue	84.54	67.63	101.45	Gamma	Cui et al. (2021)
Utility					
PFS	0.85	0.68	1.00	Beta	Li et al. (2021)
PD	0.73	0.58	0.88	Beta	Li et al. (2021)
Disutility due to grade ≥3 AEs					
Anemia	0.07	0.06	0.08	Beta	Zhu et al. (2023)
Neutrophil count decreased	0.09	0.07	0.11	Beta	Zhu et al. (2023)
Febrile neutropenia	0.13	0.10	0.16	Beta	Coyle et al. (2017)
Platelet count decreased	0.65	0.52	0.78	Beta	Zhu et al. (2023)
Diarrhea	0.21	0.17	0.25	Beta	Coyle et al. (2017)
Peripheal neuropathy	0.23	0.18	0.28	Beta	Coyle et al. (2017)
Vomit	0.13	0.10	0.16	Beta	Liu et al. (2023)
Fatigue	0.47	0.38	0.56	Beta	Coyle et al. (2017)
Risk of grade ≥3 AEs (%)					
NALIRIFOX					
Anemia	10.5	8.40	12.60	Beta	Nichetti et al. (2024)
Neutrophil count decreased	23.8	19.04	28.56	Beta	Nichetti et al. (2024)
Febrile neutropenia	2.4	1.92	2.88	Beta	Nichetti et al. (2024)
Platelet count decreased	1.6	1.28	1.92	Beta	Nichetti et al. (2024)
Diarrhea	20.3	16.24	24.36	Beta	Nichetti et al. (2024)
Peripheal neuropathy	6.8	5.44	8.16	Beta	Nichetti et al. (2024)
Vomit	7.0	5.60	8.40	Beta	Nichetti et al. (2024)
Fatigue	15.1	12.08	18.12	Beta	Nichetti et al. (2024)
FOLFIRINOX					
Anemia	11.2	8.96	13.44	Beta	Nichetti et al. (2024)
Neutrophil count decreased	30.8	24.64	36.96	Beta	Nichetti et al. (2024)
Febrile neutropenia	7.7	6.16	9.24	Beta	Nichetti et al. (2024)
Platelet count decreased	11.8	9.44	14.16	Beta	Nichetti et al. (2024)
Diarrhea	16.8	13.44	20.16	Beta	Nichetti et al. (2024)
Peripheal neuropathy	9.0	7.20	10.80	Beta	Nichetti et al. (2024)
Vomit	14.5	11.60	17.40	Beta	Nichetti et al. (2024)
Fatigue	16.5	13.20	19.80	Beta	Nichetti et al. (2024)
GEMNABP					
Anemia	18.0	14.40	21.60	Beta	Nichetti et al. (2024)
Neutrophil count decreased	34.6	27.68	41.52	Beta	Nichetti et al. (2024)
Febrile neutropenia	3.0	2.40	3.60	Beta	Nichetti et al. (2024)
Platelet count decreased	10.8	8.64	12.96	Beta	Nichetti et al. (2024)
Diarrhea	5.7	4.56	6.84	Beta	Nichetti et al. (2024)
Peripheal neuropathy	12.1	9.68	14.52	Beta	Nichetti et al. (2024)
Vomit	2.4	1.92	2.88	Beta	Nichetti et al. (2024)
Fatigue	14.5	11.60	17.40	Beta	Nichetti et al. (2024)
Body surface area (m^2^)	1.72	1.38	2.06	Gamma	Qiao et al. (2021)
Discount rate (%)	5	0	8	Fix	CGPE

OS, overall survival; PFS, progression-free survival; PD, progression disease; AE, adverse events; CGPE, china guidelines for pharmacoeconomic evaluations.

### 2.3 Cost and utility

Only direct medical costs were taken into account in this model. Biochemical examinations, blood routine tests, radiological examinations, as well as expenses related to the best supportive care and terminal care, were derived from a cost-effectiveness analysis for metastatic pancreatic cancer conducted by Na L et al. in China (Li et al., 2021). The drug price was sourced from the database of the Hunan Province Drug and Medical Consumables Procurement Management Subsystem (https://healthcare.hnybj.com.cn/), which could reflect the prices in public hospitals in China. Irinotecan, oxaliplatin, gemcitabine, and nab-paclitaxel were procured through centralized drug procurement, and their prices reflected the winning bid prices. Patients were assumed to receive subsequent treatment after disease progression. GEMNABP and FOLFOX (oxaliplatin, leucovorin, fluorouracil) were found to be the most commonly used regimens for second-line treatment (Hegewisch-Becker et al., 2019). Specifically, GEMNABP was assumed to be the second-line treatment for patients who had previously been treated with NALIRIFOX or FOLFIRINOX, while FOLFOX was employed for patients who had used GEMNABP as first-line treatment. Detailed dosing regimens were provided in Table 1. The cost related to adverse events (AEs) was gathered from a cost-effectiveness analysis for metastatic pancreatic cancer, which was based on a retrospective cohort study in China (Cui et al., 2021). The AE incidences for each treatment were derived from the NMA. Considering the relatively minimal costs and negative effects associated with grade 1–2 AEs, this model only incorporated severe AEs of grade 3 or greater. All AEs were assumed to occur in the initial cycle of the model. Costs were adjusted to the 2022 level using the Consumer Price Index and subsequently converted to U.S. dollars at an exchange rate of 1 USD to 6.7261 CNY. The utility values of PFS and PD, as well as the disutility values associated with AEs, were obtained from previously published literature, as indicated in Table 2.

### 2.4 Sensitivity analysis

One-way and probabilistic sensitivity analyses (PSA) were conducted to assess the robustness of the model. In the one-way sensitivity analysis, ranges of parameter values were based on published sources or set at ±20% of the base-case value. INMB served as a measure of economic efficiency. Second-order Monte Carlo simulation was used to perform PSA by assigning appropriate distributions to each parameter and sampling them simultaneously for 1,000 iterations. Gamma distribution was selected for costs and the body surface area, beta distribution was used for utility parameters and probabilities, as shown in Table 2.

### 2.5 Scenario analysis

#### 2.5.1 Scenario 1

According to the opinions of clinical experts, a considerable number of patients with advanced pancreatic cancer receive only best supportive care due to poor physical condition after first-line treatment, consistent with the CSCO guidelines. Therefore, we conducted a scenario analysis, assuming that patients would not receive second-line treatment after disease progression.

#### 2.5.2 Scenario 2

To reduce the toxicity of combination chemotherapy, modified versions of the FOLFIRINOX (mFOLFIRINOX) are frequently employed in clinical practice (Lambert et al., 2017). This modification involves reducing the dosage of irinotecan and omitting fluorouracil bolus. Similarly, the adjusted GEMNABP regimen is achieved by reducing the frequency of drug administration. To assess the impact of these commonly used modified or adjusted chemotherapy regimens on the model’s robustness, scenario 2 analysis was conducted. According to the recommendations of the CSCO guidelines, ajusted dosing schemes were presented in Supplementary Table S4.

#### 2.5.3 Scenario 3

To assess the potential cost-effectiveness of NALIRIFOX under current market conditions, we determined the price of liposomal irinotecan in scenario 3 by referencing the cost of the generic drug, which was $22.82/mg. Additionally, the patient assistance program (PAP) was taken into consideration despite its limitations in suitability for all patients and challenges in ensuring consistent accessibility. Discontinuing the PAP would diminish the cost-effectiveness of the regimen. The PAP for generic liposomal irinotecan enables eligible patients to receive one free dose (43 mg) of medication after purchasing one dose at their own expense. Subsequently, upon purchasing 16 doses at their own expense, patients can continue to apply for free assistance until disease progression.

### 2.6 Price simulation of liposomal irinotecan

To investigate the impact of liposomal irinotecan pricing on the cost-effectiveness of NALIRIFOX, we conducted an analysis of the variations in ICER when comparing NALIRIFOX with FOLFIRINOX or GEMNABP as the price of liposomal irinotecan gradually decreased. This analysis was carried out in both the base-case scenario and scenario 3. Additionally, a series of cost-effectiveness acceptability curves (CEACs) were developed for each treatment at various prices of liposomal irinotecan in the simulation.

## 3 Results

### 3.1 Base-case and subgroup analysis

The cost-effectiveness analysis revealed that NALIRIFOX yielded the highest LYs and QALYs, as well as the highest cost. In contrast, GEMNABP was linked to the lowest LYs, QALYs, and cost. While FOLFIRINOX produced QALYs close to those of GEMNABP, its relatively higher cost resulted in an ICER of $193,629.96/QALY, significantly higher than the WTP threshold of $38,223.34/QALY. In addition, the INMB was $-2,082.02, indicating that it was not cost-effective. Similarly, NALIRIFOX was also deemed not cost-effective due to its high cost compared to GEMNABP, as evidenced by an ICER much higher than the WTP threshold and an INMB of $-37,086.96. Overall, GEMNABP was considered the optimal choice. Additionally, based on the NMB values, the cost-effectiveness priority order was as follows: GEMNABP > FOLFIRINOX > NALIRIFOX. Details were shown in Table 3.

**TABLE 3:** Results of base-case and scenario analyses.

Strategies and scenarios	Total cost ($)	LYs	QALYs	ICER vs. GEMNABP ($/QALY)	NMB($)	INMB vs. GEMNABP ($)
Base-case analysis						
GEMNABP	9,217.58	1.01	0.58	-	13,037.05	-
FOLFIRINOX	11,811.68	1.08	0.60	193,629.96	10,955.04	−2,082.02
NALIRIFOX	50,905.5	1.09	0.70	346,330.80	−24,049.91	−37,086.96
Scenario analysis 1						
GEMNABP	6,607.07	1.01	0.58	-	15,647.57	-
FOLFIRINOX	10,964.73	1.08	0.60	325,266.60	11,801.99	−3,845.58
NALIRIFOX	50,097.77	1.09	0.70	365,821.65	−23,242.19	−38,889.76
Scenario analysis 2						
GEMNABP	9,071.66	1.01	0.58	-	13,182.98	-
FOLFIRINOX	11,215.12	1.08	0.60	159,993.13	11,551.60	−1,631.38
NALIRIFOX	50,815.75	1.09	0.70	346,797.52	−23,960.16	−37,143.14
Scenario analysis 3						
GEMNABP	9,217.58	1.01	0.58	-	13,037.05	-
FOLFIRINOX	11,811.68	1.08	0.60	193,629.96	10,955.04	−2,082.02
NALIRIFOX	21,774.45	1.09	0.70	104,318.74	5,081.13	−7,955.92

LYs, life-years; QALYs, quality-adjusted life-years; ICER, increment cost-effectiveness ratio; NMB, net monetary benefit; INMB, incremental net monetary benefit.

### 3.2 Sensitivity analysis

**FIGURE 2:**
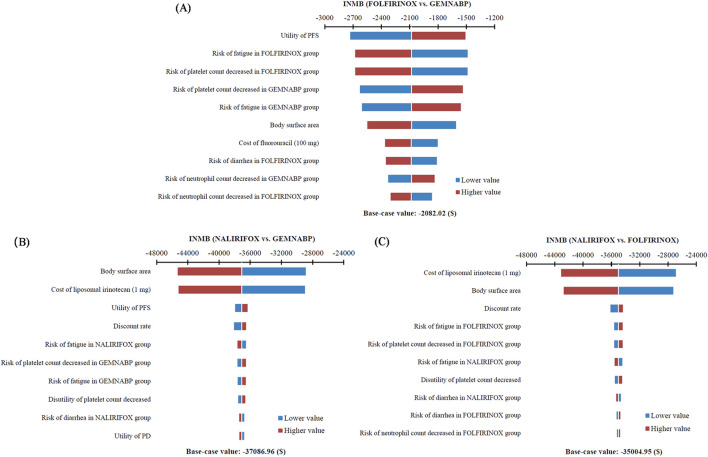
Tornado diagram of one-way sensitivity analysis. **(A)** FOLFIRINOX vs. GEMNABP. **(B)** NALIRIFOX vs. GEMNABP. **(C)** NALIRIFOX vs. FOLFIRINOX.

**FIGURE 3:**
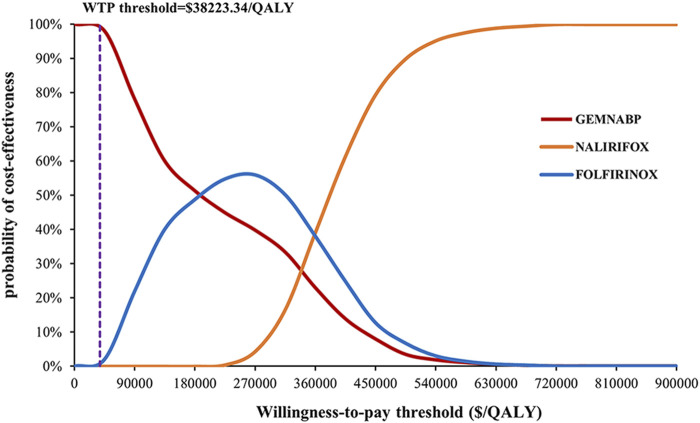
Cost-effectiveness acceptability curve of probabilistic sensitivity analysis.

The tornado diagram of one-way sensitivity analysis indicated that the results were stable, showing that both FOLFIRINOX and NALIRIFOX lacked cost-effectiveness compared to GEMNABP when the parameters varied within the defined range, as shown in Figure 2. When comparing FOLFIRINOX with GEMNABP, the model results were primarily impacted by PFS utility value, as well as the risk of AEs including fatigue and decreased platelet count. While in the analysis of the cost-effectiveness of NALIRIFOX versus GEMNABP, body surface area and the price of liposomal irinotecan were the parameters that had the greatest impact on the results. CEACs of the PSA were shown in Figure 3. With the increase of the WTP threshold, the probability that GEMNABP was more cost-effective gradually decreased. Incremental cost-effectiveness scatter plots could be found in Supplementary Figure S3.

### 3.3 Scenario analysis

The results of the scenario analysis were presented in Table 3, which align with the findings of the base-case analysis. In scenario 3, despite the lower price and more favorable PAP for generic liposomal irinotecan, NALIRIFOX was still not deemed cost-effective in comparison to GEMNABP or FOLFIRINOX.

### 3.4 Price simulation of liposomal irinotecan

**FIGURE 4:**
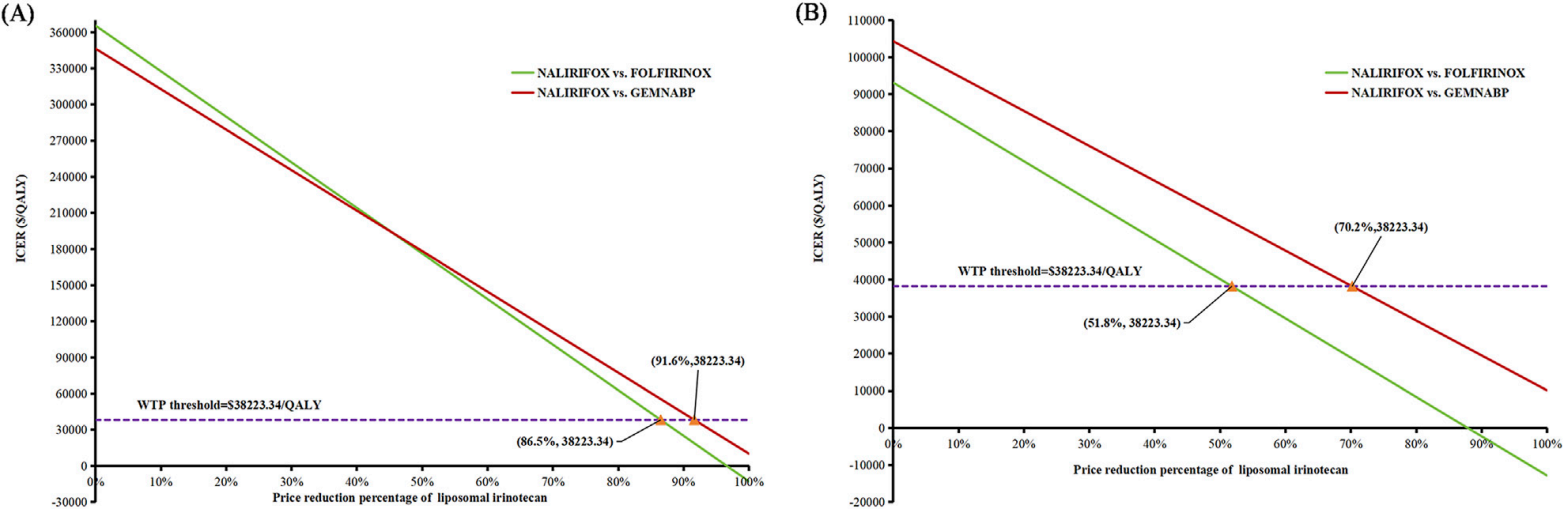
Results of price simulation of liposomal irinotecan. **(A)** Price simulation of liposomal irinotecan in base-case. **(B)** Price simulation of liposomal irinotecan in scenario 3.

**FIGURE 5:**
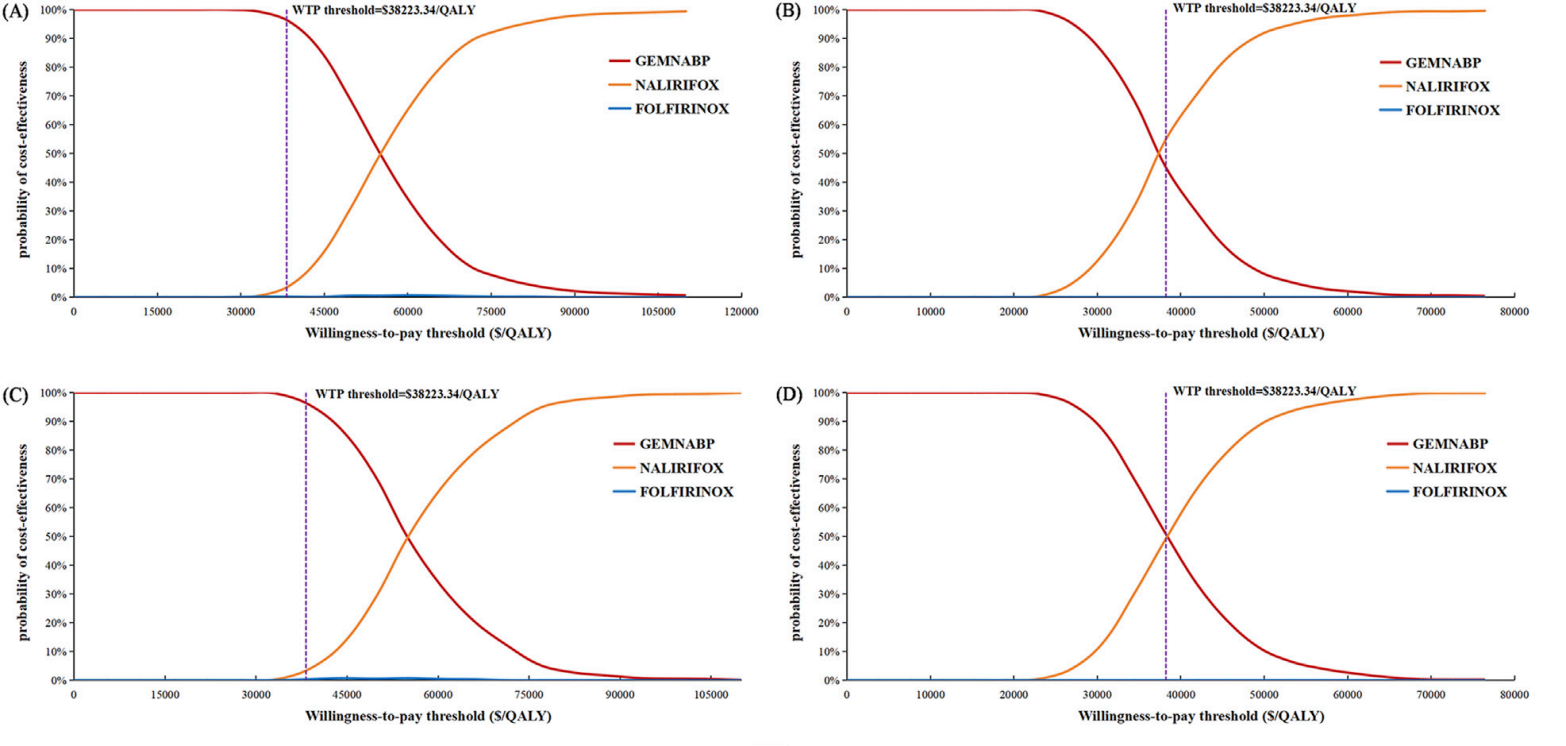
Cost-effectiveness acceptability curves in the price simulation of liposomal irinotecan. **(A)** Liposomal irinotecan is priced at $3.36/mg in base-case. **(B)** Liposomal irinotecan is priced at $2.08/mg in base-case. **(C)** Liposomal irinotecan is priced at $11/mg in scenario 3. **(D)** Liposomal irinotecan is priced at $6.81/mg in scenario 3.

Figure 4 illustrated the outcomes of the price simulation. As the price of liposomal irinotecan decreased, the ICER value of NALIRIFOX compared to GEMNABP or FOLFIRINOX displayed a gradual decline. In the base-case analysis, when the price of liposomal irinotecan decreased by more than 86.5% (less than $3.36/mg), NALIRIFOX became more cost-effective compared to FOLFIRINOX. Further, when the price reduction surpassed 91.6% (less than $2.08/mg), NALIRIFOX was more cost-effective compared to GEMNABP. In scenario 3, taking into account the PAP, NALIRIFOX became cost-effective compared to FOLFIRINOX and GEMNABP when the price reduction of generic liposomal irinotecan exceeded 51.8% (less than $11.00/mg) and 70.2% (less than $6.81/mg), respectively. The CEACs at four specific prices were presented in Figure 5. For more information on CEACs presented under a sequence of varying prices (reductions of 0%, 50%, 75%, 85%, 95%) in the base-case analysis, comparing NALIRIFOX with FOLFIRINOX or GEMNABP separately, see Supplementary Figure S4.

## 4 Discussion

When directly comparative clinical trial data is unavailable, indirect comparison and NMA is a commonly used method in health technology assessment (Kim et al., 2019). Based on previously published NMA, this study performed a cost-effectiveness analysis of three first-line combination chemotherapy regimens for metastatic pancreatic cancer. GEMNABP was associated with the lowest LYs, QALYs, and cost, whereas NALIRIFOX yielded the highest LYs, QALYs, and cost. Both FOLFIRINOX and NALIRIFOX generated ICER that was higher than the WTP threshold when compared to GEMNABP. Therefore, GEMNABP was considered the optimal choice, in line with the results of INMB. However, as NALIRIFOX and GEMNABP could gain more QALYs and LYs, comprehensive evaluation of the patient’s circumstances should be conducted when determining whether to prioritize cost-effectiveness or clinical benefits. It also should be noted that tolerance to AEs is a crucial factor influencing chemotherapy outcomes. For instance, caution should be exercised when selecting the NALIRIFOX or FOLFIRINOX for patients who have high risks of diarrhea. And when considering the risk of hematologic toxicity, NALIRIFOX poses a relatively lower risk. Although our economic evaluation model incorporated the costs and disutility values of severe AEs, clinicians are strongly encouraged to fully consider the risk of AEs when choosing treatment based on individual patient circumstances. Furthermore, upholding the patient’s autonomy in medical ethics and providing comprehensive information about potential benefits, drawbacks, and associated costs of the treatment is essential. Several investigations have reported the cost-effectiveness analysis of FOLFIRINOX versus GEMNABP. Our findings were consistent with the findings of two studies carried out in China (Zhou et al., 2016; Cui et al., 2021). On the contrary, studies conducted in Canada and the United Kingdom indicated that GEMNABP yielded lower QALYs and higher costs, making it an inferior option compared to FOLFIRINOX (Coyle et al., 2017; Gharaibeh et al., 2018; Arciero et al., 2022). The centralized drug procurement policy implemented in China may account for this phenomenon, as it has led to a notable reduction in the prices of certain drugs and an enhancement in drug affordability. For instance, the previously expensive nab-paclitaxel in GEMNABP witnessed its price drop from $401.05 in $2019 to $22.24 in 2022 after two rounds of price reductions facilitated by this policy, representing a significant decrease of 94.45%. Undoubtedly, this would have a substantial impact on the cost-effectiveness of GEMNABP. So attention should be paid to the fact that drugs in developed countries are often more expensive, and the WTP threshold for pharmacoeconomic evaluation is also higher. For example, the price of liposomal irinotecan in the United States is $62.93/mg, which is significantly higher than the price in China. The sensitivity and scenario analyses confirmed the robustness of these results.

The cost-effectiveness of NALIRIFOX was limited by its high cost. According to the tornado diagram, the body surface area and the price of liposomal irinotecan had the most substantial impact on the outcomes when comparing NALIRIFOX with GEMNABP or FOLFIRINOX, while the body surface area also affected the drug cost ultimately. Therefore, it is necessary to pay attention to the dosage and price of liposomal irinotecan in clinical application, which may have a significant impact on the cost-effectiveness of the treatment. When analyzing the cost-effectiveness of FOLFIRINOX vs. GEMNABP, the impact of AEs was more significant. The results of scenario 3 analysis indicated that, at present, the lower-priced generic drug still could not make NALIRIFOX cost-effective, even though it reduced the ICER value from $346,330.80/QALY in the base-case analysis to $104,318.74/QALY when compared to GEMNABP. Further price simulation of generic liposomal irinotecan showed that NALIRIFOX would be favored when the price was less than $11.00/mg and $6.81/mg in comparison to FOLFIRINOX and GEMNABP, respectively. However, when determining the appropriate price for the inclusion of liposomal irinotecan in the Basic Medical Insurance Drug List, it is essential to consider the impact of patients no longer being eligible for the PAP. Consequently, the drug cost should be further reduced to align with the result in the base-case analysis. NALIRIFOX would be cost-effective compared to FOLFIRINOX and GEMNABP if the price is less than $3.36/mg and $2.08/mg, respectively. Even though the required reduction is significant, the government will have a chance to achieve appropriate price through negotiation or centralized procurement when there are more manufacturers of generic drugs on the market for liposomal irinotecan. Just like the case with nab-paclitaxel. It is worth noting that although there is currently only one generic liposomal irinotecan available on the market in China, three other manufacturers’ generic drugs are already under review by the China Center for Drug Evaluation (NMPA). So revealing the potential prices of liposomal irinotecan that would make NALIRIFOX cost-effective in comparison to the other two first-line treatments for metastatic pancreatic cancer can offer valuable evidence for manufacturers and decision-makers, since the price fluctuation of liposomal irinotecan may come soon.

There are some limitations of this study. First, this cost-effectiveness analysis model was based on the NMA. The NMA incorporated phase 3 randomized controlled trials with globally comparable inclusion criteria, but heterogeneity in these trials might still affect the pooled results. For example, unlike the GEMNABP, clinical trials related to FOLFIRINOX typically had an age limit for patient enrollment, and the intervals allowed for prior adjuvant therapy to start before first-line treatment varied across studies. Therefore, it is necessary to validate and improve this model with well-designed head-to-head comparison clinical data. In addition, the clinical trials involved in the NMA primarily consist of multicenter trials conducted globally. This may not fully reflect the effectiveness and safety of these treatments in the Chinese population and potentially introduce bias to the model results. Second, utility values were collected from the literature, which may deviate from the actual data. Excluding grade 1–2 AEs when calculating the costs and utilities might result in an underestimation of real-world burden and disutility values. Nevertheless, sensitivity analyses indicated that changes in the related parameters did not alter the model results. Third, the structural uncertainty of the model, brought by the reconstruction and extrapolation of survival curves, should not be ignored. Utilizing parametric models to fit and extrapolate survival curves can introduce uncertainty in capturing complex survival hazards. Additionally, researchers may struggle to determine the best model using statistical indicators. Therefore, enhancing the model’s accuracy by validating it with long-term real-world data in the future would be highly beneficial. Fourth, while this study aimed to fully encompass the clinical application characteristics of Chinese patients, such as setting the range of body surface area at ±20% of the base-case value and considering modified versions of the FOLFIRINOX and adjusted GEMNABP regimen in scenario analysis to account for drug tolerances, it is important to acknowledge that these assumptions about patients’ demographics might not fully represent the diverse Chinese population and could introduce potential bias. Fifth, further research is required to confirm the validity of these findings in areas with existing income inequality, as the WTP threshold based on GDP *per capita* may not be applicable to certain regions. This study also overlooked potential variations between urban and rural areas within the Chinese healthcare system, which could affect cost-effectiveness.

In conclusion, the findings of this study suggested that GEMNABP was favored among these three first-line treatments thus far from an economic standpoint. Cost-effectiveness of NALIRIFOX would be obtained when the price of liposomal irinotecan was less than $3.36/mg and $2.08/mg compared to FOLFIRINOX and GEMNABP, respectively, without considering the PAP. These evidence are valuable for doctors in selecting appropriate treatment protocols and for decision-makers in determining the pricing for liposomal irinotecan. Further investigation is needed to gather additional evidence regarding the budget impact.

## Data Availability

The original contributions presented in the study are included in the article/Supplementary Material, further inquiries can be directed to the corresponding authors.
