# Supplementary material for: Cost-effectiveness analysis of first-line combination chemotherapy regimens for metastatic pancreatic cancer and evidence-based pricing strategy of liposomal irinotecan in China
Source: Front Pharmacol. 2024 Dec 20;15:1488645. doi: 10.3389/fphar.2024.1488645 (PMC11695189; doi:10.3389/fphar.2024.1488645)
Supplement: Supplementary file 1 [file DataSheet1.pdf]

Supplementary Material

1 Supplementary Tables

Supplementary Table 1. CHEERS Checklist (2022)

Topic	No.	Item	Reported?
**Title and abstract**			
Title	1	Identify the study as an economic evaluation and specify the interventions being compared.	Yes
Abstract	2	Provide a structured summary that highlights context, key methods, results, and alternative analyses.	Yes
**Introduction**			
Background and objectives	3	Give the context for the study, the study question, and its practical relevance for decision making in policy or practice.	Yes
**Methods**			
Health economic analysis plan	4	Indicate whether a health economic analysis plan was developed and where available.	Yes
Study population	5	Describe characteristics of the study population (such as age range, demographics, socioeconomic, or clinical characteristics).	Yes
Setting and location	6	Provide relevant contextual information that may influence findings.	Yes
Comparators	7	Describe the interventions or strategies being compared and why chosen.	Yes
Perspective	8	State the perspective(s) adopted by the study and why chosen.	Yes
Time horizon	9	State the time horizon for the study and why appropriate.	Yes
Discount rate	10	Report the discount rate(s) and reason chosen.	Yes
Selection of outcomes	11	Describe what outcomes were used as the measure(s) of benefit(s) and harm(s).	Yes
Measurement of outcomes	12	Describe how outcomes used to capture benefit(s) and harm(s) were measured.	Yes
Valuation of outcomes	13	Describe the population and methods used to measure and value outcomes.	Yes
Measurement and valuation of resources and costs	14	Describe how costs were valued.	Yes
Currency, price date, and conversion	15	Report the dates of the estimated resource quantities and unit costs, plus the currency and year of conversion.	Yes
Rationale and description of model	16	If modelling is used, describe in detail and why used. Report if the model is publicly available and where it can be accessed.	Yes

Topic	No.	Item	Reported?
Analytics and assumptions	17	Describe any methods for analysing or statistically transforming data, any extrapolation methods, and approaches for validating any model used.	Yes
Characterising heterogeneity	18	Describe any methods used for estimating how the results of the study vary for subgroups.	Yes
Characterising distributional effects	19	Describe how impacts are distributed across different individuals or adjustments made to reflect priority populations.	Yes
Characterising uncertainty	20	Describe methods to characterise any sources of uncertainty in the analysis.	Yes
Approach to engagement with patients and others affected by the study	21	Describe any approaches to engage patients or service recipients, the general public, communities, or stakeholders (such as clinicians or payers) in the design of the study.	Not applicable
**Results**			
Study parameters	22	Report all analytic inputs (such as values, ranges, references) including uncertainty or distributional assumptions.	Yes
Summary of main results	23	Report the mean values for the main categories of costs and outcomes of interest and summarise them in the most appropriate overall measure.	Yes
Effect of uncertainty	24	Describe how uncertainty about analytic judgments, inputs, or projections affect findings. Report the effect of choice of discount rate and time horizon, if applicable.	Yes
Effect of engagement with patients and others affected by the study	25	Report on any difference patient/service recipient, general public, community, or stakeholder involvement made to the approach or findings of the study	Not applicable
**Discussion**			
Study findings, limitations, generalisability, and current knowledge	26	Report key findings, limitations, ethical or equity considerations not captured, and how these could affect patients, policy, or practice.	Yes
Other relevant information			
Source of funding	27	Describe how the study was funded and any role of the funder in the identification, design, conduct, and reporting of the analysis	Yes
Conflicts of interest	28	Report authors conflicts of interest according to journal or International Committee of Medical Journal Editors requirements.	Yes

From: Husereau, D., Drummond, M., Augustovski, F., de Bekker-Grob, E., Briggs, A. H., Carswell, C., et al. (2022). Consolidated health economic evaluation reporting standards 2022 (CHEERS 2022) statement: Updated reporting guidance for health economic evaluations. *MDM Policy Pract.* 7(1)**,** 23814683211061097. doi:10.1177/23814683211061097

Supplementary Table 2. The median survival time of original and reconstructed Kaplan Meier survival curve.

Kaplan Meier survival curve	mPFS (95%CI)	mOS (95%CI)
original NALIRIFOX	7.4 (6.1, 7.7)	11.1(10.1, 12.3)
reconstructed NALIRIFOX	7.39 (6.06, 7.86)	11.2 (10.33, 12.2)
original FOLFIRINOX	7.3 (6.5, 7.9)	11.7 (10.4, 13)
reconstructed FOLFIRINOX	7.33 (6.48, 7.96)	11.8 (10.62, 13.2)
original GEMNABP	5.7 (5.6, 6.1)	10.4 (9.8, 10.8)
reconstructed GEMNABP	5.79 (5.68, 6.18)	10.5 (9.84, 11)

mPFS, median progression-free survival; mOS, median overall survival.

Supplementary Table 3. Summary of the statistical goodness-of-fit of Kaplan Meier survival curves.

	**Exponential**	**Weibull**	**Gamma**	**Generalized gamma**	**Gompertz**	**Log-normal**	**Log-logistic**
OS curve							
NALIRIFOX							
AIC	1,906.910	1,891.873	1,891.413	1,893.390	1,897.522	1,900.535	1,898.422
BIC	1,910.858	1,899.770	1,899.309	1,905.234	1,905.418	1,908.431	1,906.318
FOLFIRINOX							
AIC	2,180.960	2,148.930	2,151.031	2,150.820	2,157.560	2,195.154	2,166.546
BIC	2,185.031	2,157.072	2,159.173	2,163.033	2,165.702	2,203.296	2,174.687
GEMNABP							
AIC	9,429.609	9,243.105	9,235.604	9,237.604	9,323.104	9,332.456	9,271.968
BIC	9,435.085	9,254.056	9,246.556	9,254.032	9,334.056	9,343.408	9,282.920
PFS curve							
NALIRIFOX							
AIC	1,719.482	1,705.557	1,704.336	1,705.994	1,713.135	1,721.002	1,713.021
BIC	1,723.684	1,713.962	1,712.741	1,718.602	1,721.540	1,729.407	1,721.426
FOLFIRINOX							
AIC	1,730.826	1,706.838	1,704.463	1,705.557	1,719.704	1,713.208	1,712.889
BIC	1,734.896	1,714.979	1,712.605	1,717.769	1,727.846	1,721.350	1,721.030
GEMNABP							
AIC	7,517.293	7,316.263	7,299.780	7,300.345	7,414.356	7,384.377	7,336.912
BIC	7,522.769	7,327.215	7,310.732	7,316.773	7,425.308	7,395.329	7,347.863

OS, overall survival; PFS, progression-free survival; AIC, Akaike's information criterion; BIC, Bayesian information criterion.

Supplementary Table 4. Specific dosing regimens of modified chemotherapy regimen.

Modified chemotherapy regimen	Dosing schemes
mFOLFIRINOX	Irinotecan 150 mg/m2 + oxaliplatin 85 mg/m2 + LV 400 mg/m2 +fluorouracil 2400 mg/m2 over 46h; every 14 days
mGEMNABP	GEM 1000 mg/m2 + NABP 125 mg/m2; Days 1, 8, and every 21 days

GEM, gemcitabine; LV, leucovorin; NABP, nab-paclitaxel.

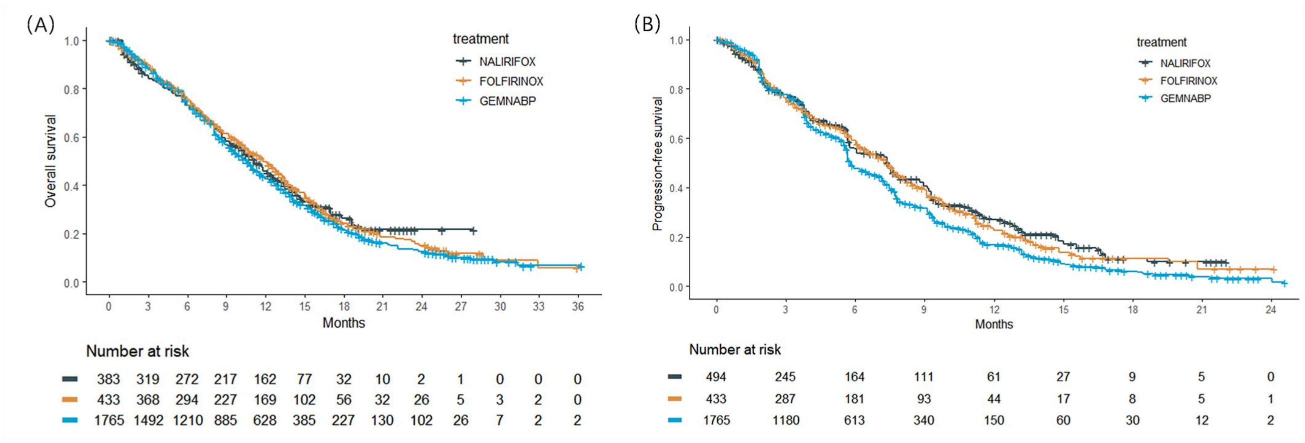

Supplementary Figure 1. Reconstruction of Kaplan Meier survival curve. (A) OS curve. (B) PFS curve. OS, overall survival; PFS, progression-free survival.

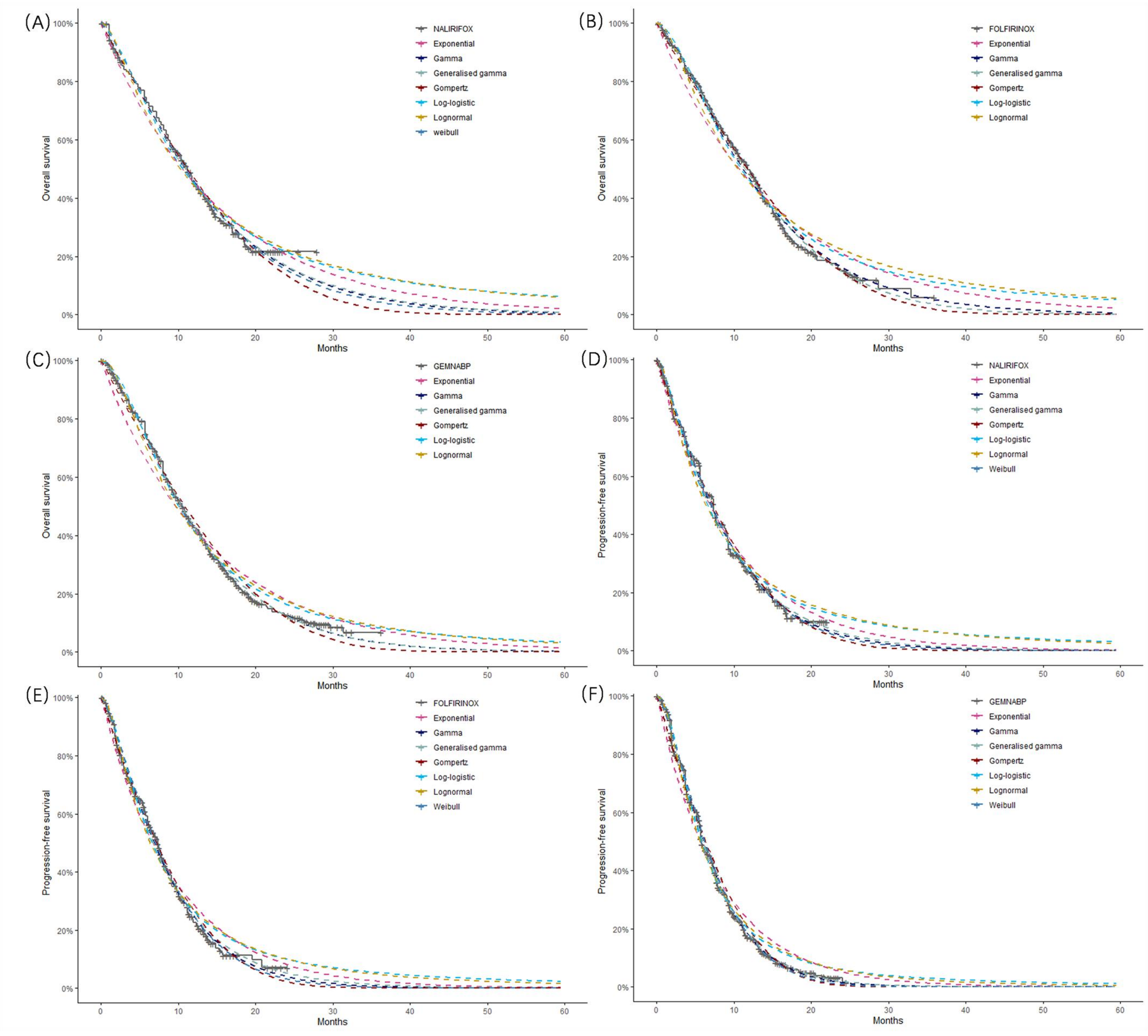

Supplementary Figure 2. Fitting and extrapolation of Kaplan Meier survival curve. (A) The results of NALIRIFOX OS curve. (B) The results of FOLFIRINOX OS curve. (C) The results of GEMNABP OS curve. (D) The results of NALIRIFOX PFS curve. (E) The results of FOLFIRINOX PFS curve. (F) The results of GEMNABP PFS curve. OS, overall survival; PFS, progression-free survival.

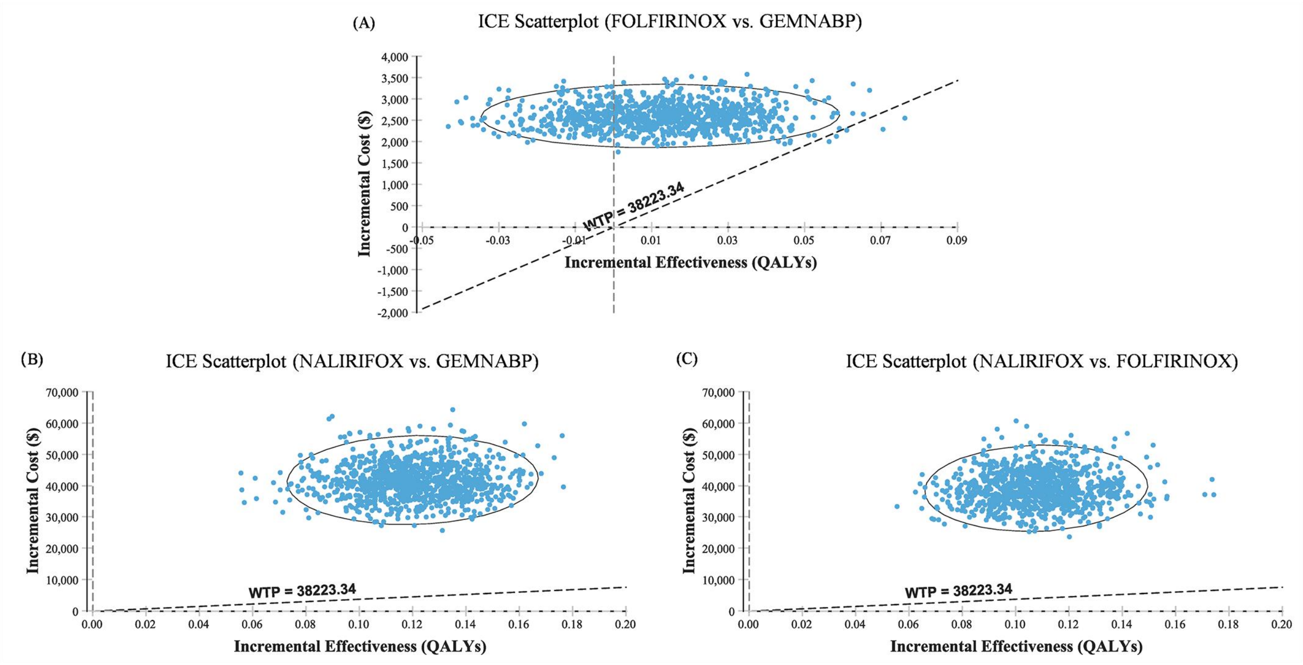

Supplementary Figure 3. Incremental cost-effectiveness scatter plots. (A) FOLFIRINOX vs. GEMNABP. (B) NALIRIFOX vs. GEMNABP. (C) NALIRIFOX vs. FOLFIRINOX. ICE, incremental cost-effectiveness.

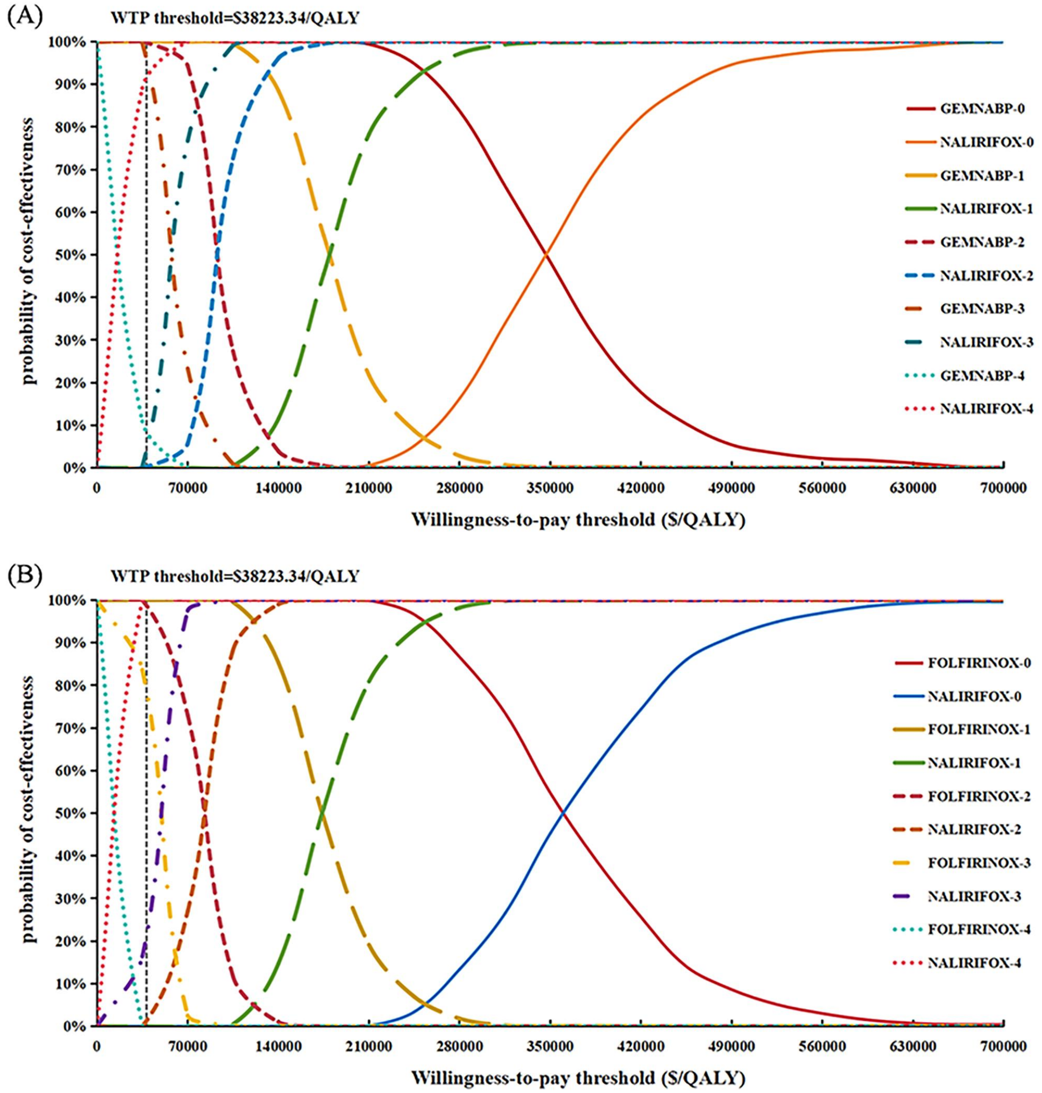

Supplementary Figure 4. Cost-effectiveness acceptability curves conducted under a sequence of varying prices in the base-case. 0-4 refer to the price reductions of 0%, 50%, 75%, 85%, 95%.
